# Electrochemical and Capacitive Properties of Carbon Dots/Reduced Graphene Oxide Supercapacitors

**DOI:** 10.3390/nano6110212

**Published:** 2016-11-14

**Authors:** Yong-Qiang Dang, Shao-Zhao Ren, Guoyang Liu, Jiangtao Cai, Yating Zhang, Jieshan Qiu

**Affiliations:** 1 College of Chemistry and Chemical Engineering, Xi’an University of Science and Technology, No. 58 Yanta Road, Xi’an 710054, China; ddyqng@163.com (Y.-Q.D.); renshaozhao@126.com (S.-Z.R.); liuguoyangxust@126.com (G.L.); caijiangtao11@gmail.com (J.C.); 2 State Key Lab of Fine Chemicals, Liaoning Key Lab for Energy Materials and Chemical Engineering, PSU-DUT Joint Center for Energy Research, Dalian University of Technology, No. 2 Ling Gong Road High Technology Zone, Dalian 116024, China

**Keywords:** carbon dots, supercapacitors, graphene

## Abstract

There is much recent interest in graphene-based composite electrode materials because of their excellent mechanical strengths, high electron mobilities, and large specific surface areas. These materials are good candidates for applications in supercapacitors. In this work, a new graphene-based electrode material for supercapacitors was fabricated by anchoring carbon dots (CDs) on reduced graphene oxide (rGO). The capacitive properties of electrodes in aqueous electrolytes were systematically studied by galvanostatic charge-discharge measurements, cyclic voltammetry, and electrochemical impedance spectroscopy. The capacitance of rGO was improved when an appropriate amount of CDs were added to the material. The CD/rGO electrode exhibited a good reversibility, excellent rate capability, fast charge transfer, and high specific capacitance in 1 M H_2_SO_4_. Its capacitance was as high as 211.9 F/g at a current density of 0.5 A/g. This capacitance was 74.3% higher than that of a pristine rGO electrode (121.6 F/g), and the capacitance of the CD/rGO electrode retained 92.8% of its original value after 1000 cycles at a CDs-to-rGO ratio of 5:1.

## 1. Introduction

Because of the rapidly increasing demands for energy storage devices in electric vehicles, mobile electronic devices, and memory backup systems, supercapacitors—which have high power densities, fast charge-discharge rates, and long lifetimes [1,2,3]—have been the topic of much research interest. Supercapacitors, or electrochemical capacitors, generally consist of four parts: an electrode, an electrolyte, a collector, and a separator. The electrode material is one of the most important factors influencing a supercapacitor’s electrochemical properties. In recent years, graphene, which is composed of two-dimensional layers of sp^2^ bonded carbon [4], has been shown to be a promising electrode material because of its unique structure and properties, such as its large specific surface area, high electrical conductivity, and good chemical stability [5,6,7]. However, the strong π-π interactions between graphene sheets generated during their synthesis result in the agglomeration of these sheets, reducing the size of the pores that are required for allowing the access of electrolytes [8] and decreasing the specific capacitances of the resulting graphene electrodes. To overcome this problem, graphene is often used in composite materials, such as conducting polymer/graphene composite electrodes (e.g., reduced graphene oxide (rGO)/polypyrrole [9], rGO/polyaniline [10], and graphene/3,4-Ethylenedioxythiophene [11]), graphene/metal oxide composite electrodes (e.g., 33D-graphene/α-MnO_2_ nanofibers [12]), and hetero atom-doped graphene composite electrodes [13].

Carbon dots (CDs), mainly including graphene quantum dots (GQDs), carbon nanodots (CNDs), and polymer dots (PDs) [14], are quasi-zero-dimensional nanomaterials with a high water solubility, high chemical stability, simple surface functionalization chemistry, and excellent electro-optical characteristics [15,16], making them promising materials for the design and fabrication of novel devices in the multidisciplinary fields of photocatalysis [17,18], chemical sensing [19], electrocatalysis [20], biosensing [21], and supercapacitors [22]. Recently, as one type of CDs, graphene quantum dots (GQDs) have been effectively improving supercapacitors’ performances in the electrochemical capacitive properties of GQDs supercapacitors [23], GQDs/MnO_2_ supercapacitors [24], GQDs/polyaniline nanofiber supercapacitors [25], and GQDs/polyaniline asymmetric micro-supercapacitors [26]. These studies provide new insight for increasing specific capacitances of the electrode materials for high-performance supercapacitors. In this article, CDs were used to reduce the agglomeration of graphene sheets, which may increase the specific capacitances of rGO electrodes.

Herein, we report the facile fabrication of CDs/graphene composites via the covalent bond for supercapacitors. The addition of an appropriate amount of CDs significantly enhanced the specific electrochemical capacitance. A composite electrode with a CDs-to-rGO ratio of 5:1 displayed a good reversibility, an excellent rate capability, fast charge transfer, and a specific capacitance of 211.9 F/g, which was 74.3% higher than that of rGO electrode (121.6 F/g). These encouraging results suggest that CDs/rGO supercapacitors have high performances.

## 2. Results and Discussion

**Figure 1:**
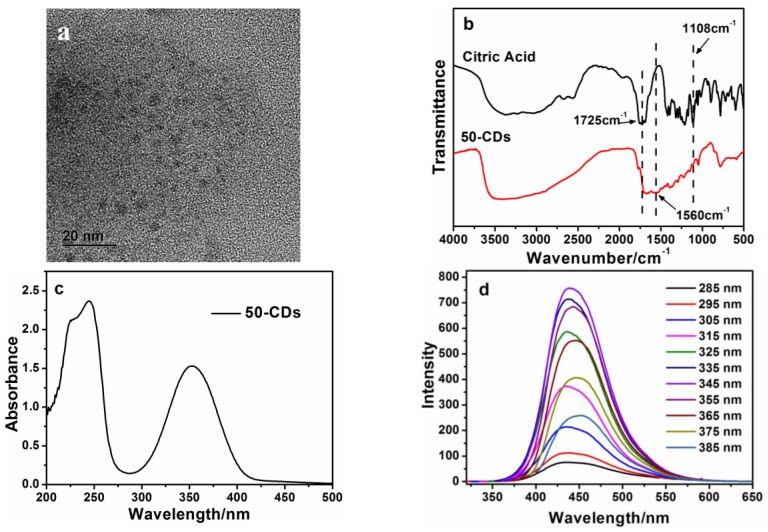
(**a**) Transmission electron microscopy (TEM) of the 50-carbon dots (CDs); (**b**) Fourier transform infrared (FTIR) spectra of citric acid and the 50-CDs; (**c**) Ultraviolet (UV)-visible absorption spectrum of the 50-CDs; (**d**) Photoluminescence (PL) spectra of the 50-CDs at different excitation wavelengths.

The CDs were synthesized through a hydrothermal method according to the article published by Zhu et al. [27]. Briefly, a solution of citric acid and ethylenediamine with a certain proportion were heated in a Teflon-lined autoclave at 180 °C for 5 h. Then CDs powder was obtained after dialysis and freeze-drying. The CDs were distinguished by the molar fraction of citric acid used in raw materials. For example, 50-CDs means that these CDs were synthesized using a citric acid mole fraction of 50%. The morphologies of the as-prepared 50-CDs were investigated with transmission electron microscopy (TEM). As shown in Figure 1a, the 50-CDs were spherically shaped with the size in the range of 3 nm–5 nm. According to the Fourier transform infrared (FTIR) analysis of 50-CDs (Figure 1b), an N–H bending vibration was observed at 1560 cm^−1^ for the 50-CDs, indicating that amino functional groups were successfully formed on their surfaces. Meanwhile, the C=O vibrational absorption band at 1725 cm^−1^ for citric acid was very low in the 50-CDs’ spectra. The C–O and –OH stretching vibrations at 1108 cm^−1^ and 1410–1260 cm^−1^, respectively, were also diminished in the 50-CDs. These results indicate that the oxygen-containing groups on the citric acid-modified surfaces were reduced during the hydrothermal process because of condensation and carbonization reactions [27]. The Ultraviolet (UV)-visible absorption spectra (Figure 1c) contained a clear absorption band near 350 nm, which was similar to that reported by Zhu et al. [27]. Figure 1d shows the photoluminescence (PL) spectra of the 50-CDs at different excitation wavelengths. The PL peaks at 430 nm were red-shifted to ca. 450 nm when the excitation wavelength changed from 285 nm to 385 nm. This result revealed that the 50-CDs prepared here also had an excitation-dependent PL behavior [28], which was contributed to the surface state affecting the band gap of 50-CDs [27]. From the TEM, UV-Visible absorption spectra, and PL spectra results, it can be concluded that 50-CDs were synthesized successfully.

**Figure 2:**
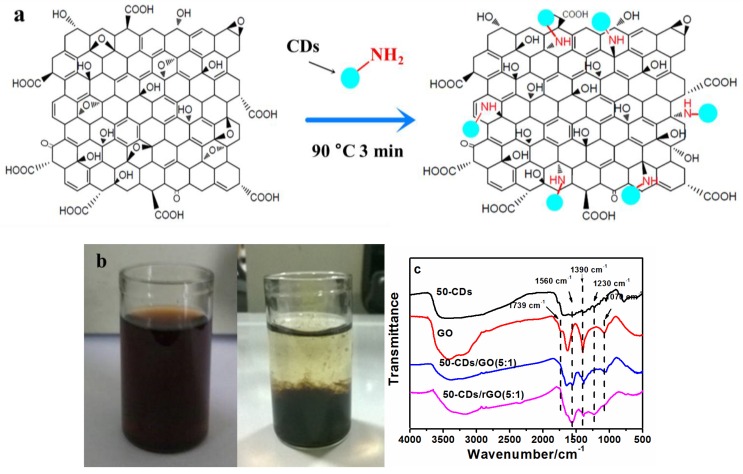
(**a**) Depiction of the CDs/graphene oxide (GO) synthesis reaction; (**b**) Mixed aqueous solution of GO and 50-CDs before (left) and after (right) heat treatment; (**c**) FTIR spectra of the 50-CDs, GO, 50-CDs/GO (5:1), and 50-CDs/rGO (5:1).

**Figure 3:**
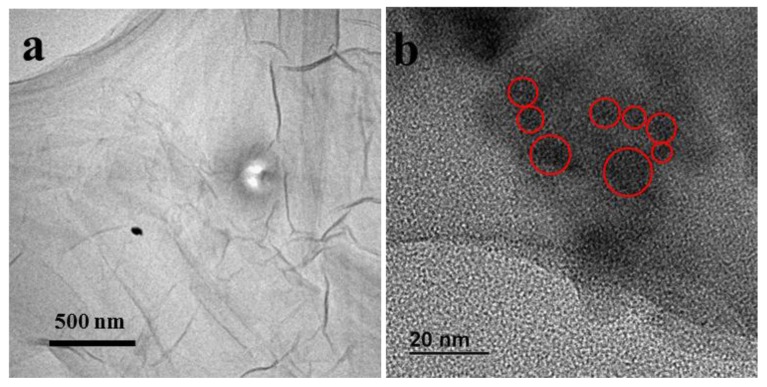
TEM image of (**a**) GO, and (**b**) CDs/rGO (5:1), some CDs are circled in red.

To fabricate the CDs/GO composite, CDs were added to GO aqueous solution and heated at 90 °C for 3 min. During this process, amino functional groups on the surfaces of the CDs reacted with epoxy groups on GO via a ring-opening reaction [29,30], which resulted in the anchoring of the CDs to the GO sheets via their oxygen-containing functional groups, as shown in Figure 2a. The mass ratio of 50-CDs to GO using synthesis was placed in round brackets at the end of composite. After this reaction, the unreacted amino functional groups on CDs were free to react with other GO sheets, causing precipitation in the aqueous solution (Figure 2b). Subsequently, the CDs/GO composite was reduced via a reduction reaction in hydrogen dielectric barrier discharge (DBD) plasma to produce the CDs/rGO composite, written CDs/rGO (5:1), in order to improve graphene’s electrical conductivity [31,32,33]. FTIR spectroscopy was used to characterize the reaction and reduction processes, as shown in Figure 2c. According to the FTIR spectra, the C–N stretching vibration was observed at 1230 cm^−1^ in 50-CDs/GO (5:1) and 50-CDs/rGO (5:1) after the ring-opening reaction, while the stretching vibration of C–O–C at 1070 cm^−1^ was weakened in 50-CDs/GO (5:1) and 50-CDs/rGO (5:1). These results indicated that the CDs were successfully anchored on the GO. Meanwhile, the C=O stretching vibration at 1739 cm^−1^ and the O–H bending vibration at 1390 cm^−1^ were observed in GO and 50-CDs/GO (5:1), but these bands were weaker in 50-CDs/rGO (5:1). A band at 1560 cm^−1^ and a small band near 1439 cm^−1^ were observed in the 50-CDs/GO (5:1) spectra, which might have been due to the presence of an amide. These peaks were increased in 50-CDs/GO (5:1), perhaps because of contributions from the antisymmetric stretching vibration of C=C. These results indicated that the oxygen-containing groups on graphene were reduced in the hydrogen plasma. TEM images of GO and 50-CDs/rGO (5:1) are shown in Figure 3. From the TEM image shown in Figure 3a, it can be seen that GO exhibits semitransparent thin sheet with rich wrinkles. In Figure 3b, CDs (red circle) aggregates due to the amino groups and a small number of carboxyl group existing on CD surface, and CDs are mixed up with rGO (Figure 3b), which also indicate that the CDs/rGO (5:1) composite formed.

**Figure 4:**
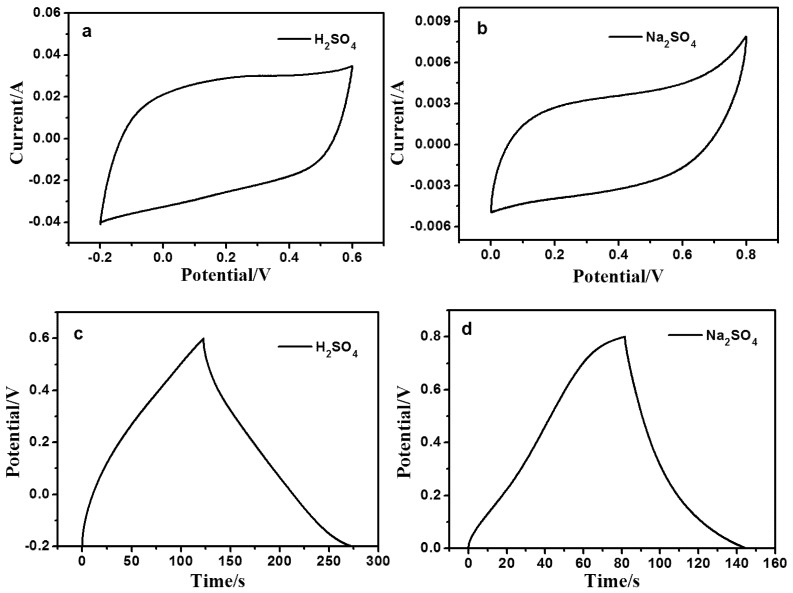
(**a**) Cyclic voltammetry (CV) curves of 50-CDs/rGO (5:1) in 1 M H_2_SO_4_ at a scan rate of 0.2 V/s; (**b**) CV curves of 50-CD/rGO (5:1) in 1 M Na_2_SO_4_ at a scan rate of 0.2 V/s; (**c**) Galvanostatic charge-discharge (GCD) curves of 50-CDs/rGO (5:1) in 1 M H_2_SO_4_ at current density of 1 A/g; (**d**) GCD curves of 50-CDs/rGO (5:1) in 1 M Na_2_SO_4_ at current density of 1 A/g.

The electrolyte is important for determining the characteristics of a supercapacitor. In this work, 1 M H_2_SO_4_ and 1 M Na_2_SO_4_ were used as electrolytes, and their electrochemical behaviors were evaluated using cyclic voltammetry (CV) and galvanostatic charge-discharge (GCD) measurements. As shown in Figure 4a,b, the CV curves of the 50-CDs/rGO (5:1) electrode in 1 M H_2_SO_4_ electrolyte were similar to ideal electrochemical double layer behavior [34], without obvious oxygen and hydrogen evolution peaks. The area under the CV curve produced in 1 M H_2_SO_4_ was larger than that produced in 1 M Na_2_SO_4_. Assuming that the specific capacitance has a direct relationship with the specific current at a given scan rate, a larger area under the curve indicates that a supercapacitor has a larger specific capacitance [35]. As shown in Figure 4c,d, the discharge times of the GCD curves in 1 M H_2_SO_4_ were longer than those in 1 M Na_2_SO_4_, confirming that H_2_SO_4_ is a superior electrolyte and improved the specific capacitance of this supercapacitor. For this reason, the remaining electrochemical tests were performed in 1 M H_2_SO_4_. Therefore, 1 M H_2_SO_4_ were used as electrolytes in all subsequent experiments.

**Figure 5:**
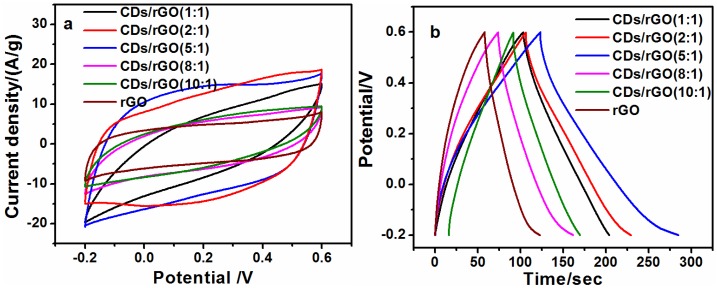
(**a**) CV curves of CDs/rGO (1:1), CDs/rGO (2:1), CDs/rGO (5:1), CDs/rGO (8:1), CDs/rGO (10:1), and rGO at a scan rate of 0.2 V/s; (**b**) GCD curves of CDs/rGO (1:1), CDs/rGO (2:1), CDs/rGO (5:1), CDs/rGO (8:1), CDs/rGO (10:1), and rGO at a current density of 0.5 A/g.

Different 50-CDs/rGO composites were prepared by adjusting the mass ratio of 50-CDs to GO during synthesis, and then CV and GCD measurements were performed on them in order to evaluate whether the CDs/rGO composites have higher specific capacitances than rGO. As shown in Figure 5a, the CV curves of all of the 50-CDs/rGO electrodes and the rGO electrode exhibited a relatively ideal rectangular shape, and no obvious redox peaks were observed through the entire scan range, indicating ideal electric double-layer capacitance behaviors [36]. The area under the CV curve of each CDs/rGO electrode is larger than that of rGO electrode, especially the 50-CDs/rGO (5:1), in which the mass ratio of CDs to rGO is 5:1, is the largest one. Such results imply that CDs/rGO (5:1) possess the highest specific capacitance. The GCD curves of all of the electrodes at 0.5 A/g are shown in Figure 5b. No apparent potential drop was observed at the onset of GCD measurements, revealing that the electrodes had low internal resistances, and the discharging time of each CDs/rGO electrode is nearly longer than that of rGO, the CDs/rGO (5:1) has the longest discharging time. To compare their specific capacitances quantitatively, the specific capacitances of the rGO and various CDs/rGO electrodes were calculated from the GCD curves at different current densities of 0.5 A/g, 1 A/g and 2 A/g (Supplementary Materials Table S1). The values also indicate that nearly all of the CDs/rGO electrodes had a higher specific capacitance than rGO electrodes. These results suggested that the presence of CDs improved the specific capacitances of rGO, and the CDs/rGO at a 5:1 CDs-to-rGO mass ratio had the highest specific capacitance. More CDs will result in the reduction of specific capacitance, which can be attributed to lower surfaces of CDs than rGO.

**Figure 6:**
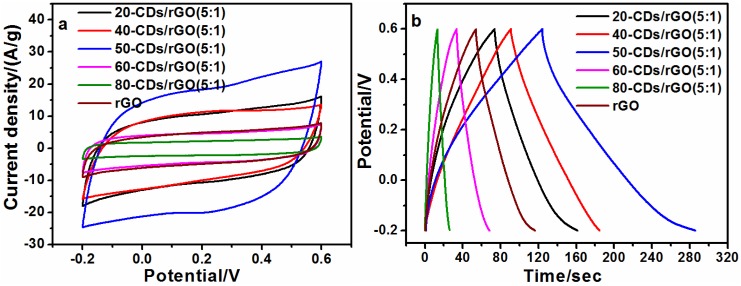
(**a**) CV curves of the 20-CDs/rGO, 40-CDs/rGO, 50-CDs/rGO, 60-CDs/rGO, 80-CDs/rGO and rGO electrodes at a scan rate of 0.2 V/s; (**b**) GCD curves of the 20-CDs/rGO, 40-CDs/rGO, 50-CDs/rGO, 60-CDs/rGO, 80-CDs/rGO and rGO electrodes at a current density of 1 A/g.

Since tuning the reaction conditions will lead to CDs having different PL properties [27], we synthesized CDs under different citric acid-to-ethylenediamine molar ratio, in order to investigate if the specific capacitance of CDs/rGO composites can be affected. The number before “CDs” denotes the mole fraction of citric acid during synthesis, and the mass ratio of CDs to rGO is 5:1 in this part. Figure 6a shows CV curves of rGO and CDs/rGO electrodes prepared with different CDs. In the figure, the area (S) under the CV curve of each electrode increased in the order S_80-CDs/rGO_ < S_60-CDs/rGO_ < S_rGO_ < S_20-CDs/rGO_ < S_40-CDs/rGO_ < S_50-CDs/rGO_. Such results demonstrated that the CDs did affect the specific capacitance of CDs/rGO composites, and 50-CDs/rGO possessed the highest specific capacitance. These results were also confirmed by GCD curves (Figure 6b) and their calculated specific capacitances at different current densities (Supplementary Materials Table S1). The results accorded with the order obtained from CV curves. The citric acid acting as the main carbon source of CDs and supporting the reactive site to react with ethylenediamine, the concentration of citric acid used during synthesis plays an important role in tuning the surface properties of CDs. The amino group on the CDs is the main functional group to react with GO. If the amount of amino group is too less, the rGO sheets cannot be separated well, which result in lower specific capacitance at lower concentration of ethylenediamine. If the amount of amino group is too more, one CD particle with many amino groups might be wrapped by one rGO sheet, which lead to the decrease of rGO surface area. Then the specific capacitance decreases. From the results of Figure 6, the specific capacitance is the highest when the molar ratio of citric acid to ethylenediamine is 1:1. Combining the results in Figure 5 and Figure 6, a conclusion can be drawn that CDs can be used to improve the specific capacitance of rGO with a proper type and a proper fraction.

**Figure 7:**
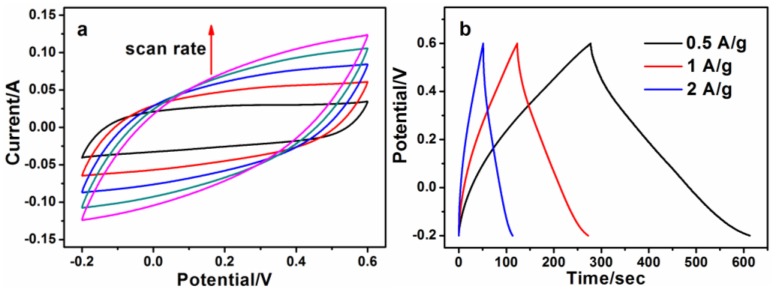
(**a**) CV curves of 50-CDs/rGO (5:1) at different scan rates of 0.2, 0.4, 0.6, 0.8 and 1 V/s, respectively, where the arrow indicates the direction of increasing scan rates; (**b**) GCD curves of 50-CDs/rGO (5:1) at different current densities of 0.5 A/g, 1 A/g and 2 A/g, respectively.

Then, the electrochemical and capacitive properties of the best composite, 50-CDs/rGO (5:1), were studied in detail. Firstly, the CV curve was measured under different scan rates (Figure 7a). It shows that the CV curve remained quasi-rectangular as the scan rate increased (Figure 7a), demonstrating the excellent reversibility and good capacitive properties of the 50-CDs/rGO (5:1) electrode. Moreover, the response current increased with an increase in scan rate, suggesting an excellent rate capability of 50-CDs/rGO (5:1). As shown in Figure 7b, the GCD curves of the 50-CDs/rGO (5:1) electrode at different current densities were approximately triangle-shaped, suggesting the capacitors had high reversibilities and ideal capacitor behaviors. These features mainly originated from the electric double layer at the electrode/electrolyte interface of the 50-CDs/rGO (5:1) electrode [37].

**Figure 8:**
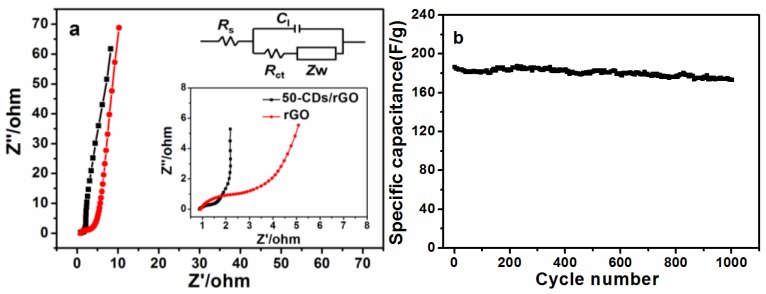
(**a**) Nyquist impedance plot of the rGO and 50-CDs/rGO electrodes. Inset is an expanded view in the region of high frequencies and an equivalent circuit model; (**b**) the specific capacitance of the 50-CDs/rGO supercapacitor for charging/discharging as a function of cycle number measured at a current density of 1 A/g.

Electrochemical impedance spectroscopy (EIS) was performed to further evaluate the electrochemical performances of the different materials, as shown in Figure 8a. The experimental data were fitted by an equivalent circuit shown in the inset of Figure 8a, where the *R*_s_ is the equivalent series resistance, *R*_ct_ is the charge transfer resistance, *Z*w is the Warburg impedance, *C*_l_ is the electric double capacitance. The impedance spectrum of an ideal electrical double-layer capacitor (EDLC) contains a depressed semicircle in the high-frequency region and straight line in the low-frequency region [38,39]. As the Nyquist plot shown in Figure 8a, both the straight lines of 50-CDs/rGO and rGO in the low-frequency region were nearly parallel to the imaginary impedance axis, implying ideal capacitive behaviors. In the high-frequency region, the intersection point of the arc and the real impedance axis is the *R*_s_, which is the sum of the electrolyte solution resistance, the intrinsic resistance of the active material, and the resistance at the electrode/electrolyte interface [40]. As shown in a higher resolution view of the high-frequency region (inset to Figure 8a), the *R*_s_ values of both electrodes are similar, suggesting that both the rGO and 50-CDs/rGO electrodes had low electrode/electrolyte interfacial resistances [41]. In addition, the diameter of the arcs is an indicator of *R*_ct_ combined with the double-layer capacitance between the active electrode material and the electrolyte [42]. The EIS spectra were fitted to the equivalent circuit model shown in the inset to Figure 8a. In the equivalent circuit, the *R*_ct_ value of the 50-CDs/rGO electrode (0.82 Ω) was much lower than that of the rGO electrode (2.73 Ω). This indicated that the added CDs enabled more rapid charge transfer at the electrode/electrolyte interface [23,43]. Figure 8b shows the capacitance retention rate of the 50-CD/rGO electrode at a current density of 1 A/g. The 50-CDs/rGO electrode retained 92.8% of its original capacitance in 1 M H_2_SO_4_ after 1000 cycles. The excellent cycle stability of the 50-CDs/rGO electrode implied stable energy-storage processes during long cycle charging/discharging.

## 3. Materials and Methods

### 3.1. Chemicals

Citric acid, ethylenediamine, sulfuric acid and sodium sulfate were purchased from Sinopharm Chemical Reagent Co., Ltd. (Beijing, China). Aqueous solutions of graphene oxide (GO) were purchased from Hengqiu Graphene Technology Co. Ltd. (Suzhou, China). All reagents were used without further purification.

### 3.2. Characterization

Transmission electron microscopy (TEM, HITACHI H-600, Tokyo, Japan) were performed to characterize the structural characteristics and morphologies of the as-prepared samples. Photoluminescence (PL) spectra were obtained using a fluorescence spectrophotometer (Perkin-Elmer LS-55, Waltham, MA, USA). UV-visible spectra were obtained using a UV-visible spectrophotometer (Thermo-Scientific Evolution-220, Waltham, MA, USA). Fourier transform infrared (FTIR) spectra were recorded with a Perkin-Elmer Spectrum GX FTIR Spectrometer (Waltham, MA, USA). All electrochemical tests, including galvanostatic charge-discharge (GCD), cyclic voltammetry (CV), and electrochemical impedance spectroscopy (EIS), were carried out using a CHI-660D electrochemical workstation (ChenHua, Shanghai, China).

### 3.3. Preparation of CDs

CDs were synthesized through a hydrothermal method [27]. Briefly, 0.84 g of citric acid and 1 mL of ethylenediamine solution (1:4 molar ratio of citric acid to ethylenediamine) were added to 20 mL of deionized water. The mixture was stirred, and the resulting suspension was transferred to a Teflon-lined autoclave and heated at 180 °C for 5 h. After being cooled to room temperature, the water-soluble CDs were purified by dialysis for about 24 h using a dialysis tube (1000 Da molecular weight cutoff). Finally, the CDs powder (20-CDs) was obtained by freeze-drying the sample. Four other types of CDs powders were prepared by controlling the amount of citric acid used in the process. These samples are referred to as 40-CDs, 50-CDs, 60-CDs and 80-CDs, in which the mole fraction of citric acid is 40%, 50%, 60% and 80%, respectively.

### 3.4. Fabrication of CD/rGO

A 3 mL GO aqueous solution (2 mg/L) was sonicated for 30 min. 50-CDs (6 mg) was then added to this solution, which was subsequently sonicated for 10 min. The mixture was heated in a water bath at 90 °C for about 3 min. Then the mixture was centrifuged, and the supernatant was removed. After being washed with deionized water three times, the mixture was freeze-dried. Then the target CDs/rGO (1:1) powder was obtained by reducing CDs/GO (1:1) using a hydrogen dielectric barrier discharge (DBD) plasma technique [44]. Typically, GO or CDs/GO powder was put into a plate quartz tube (about 60 mm in inner diameter, 110 mm in outside diameter, and 8 mm high). The reactor was purged with H_2_ to exhaust the air in the quartz tube firstly. Then the DBD plasma was initiated under 50 V × 1.2 A input ac power at room temperature and atmospheric pressure for 25 min using a low temperature plasma power supply (Coronalab CTP-2000K/P, Nanjing, China). The same process was performed at four different mass ratios of 2:1, 5:1, 8:1 and 10:1 CDs/rGO using the 50-CDs. The mass ratio of CDs to GO after reaction and reduction was not measured; their ratio during synthesis was just used to describe them. Pure rGO was also prepared without adding CDs. In addition, the four other types of CDs were also used to fabricate CDs/rGO at a 5:1 CDs-to-rGO mass ratio.

### 3.5. Electrochemical Measurements

For the electrochemical measurement, the working electrode was prepared as follows. Typically, the electroactive materials were mixed up with carbon black and polyvinylidene fluoride (PVDF) binder in a mass ratio of 8:1:1 and dispersed in ethanol, and the resulting mixture was dried at 85 °C. After drying, it was pressed into a wafer of 10 mm diameter and assembled into an electrode with two pieces of titanium mesh (12 mm in diameter).

The electrochemical properties of rGO or CDs/rGO were measured in a standard three-electrode system with a Pt sheet as counter electrode and saturated calomel electrode as reference electrode in 1 M H_2_SO_4_ or 1 M Na_2_SO_4_ aqueous electrolyte.

Gravimetric capacitance was calculated from GCD curves using Equation (1):

*C* = *Q*/(*m* × ∆*U*) = *It*/(*m* × ∆*U*), (1)

where *C* is the specific capacitance (F/g), *I* is the discharge current (A), *t* is the discharge time (s), ∆*U* is the discharge potential window (V), and *m* is the mass of active material (g).

## 4. Conclusions

CDs were successfully anchored on GO in a water bath at 90 °C for 3 min, and CDs/rGO was synthesized via the reduction of these materials in a hydrogen plasma. The electrochemical performances of the synthesized composites were studied, and CDs/rGO was found to be an effective supercapacitor. The CDs/rGO supercapacitors exhibited high specific capacitances in 1 M H_2_SO_4_. Additionally, compared with other CDs/rGO and rGO supercapacitors, the 50-CDs/rGO supercapacitor exhibited the best reversibility, fastest charge transfer, and highest specific capacitances of 211.9 F/g, 186.5 F/g and 155.3 F/g at 0.5 A/g, 1 A/g and 2 A/g, respectively. In cycling tests, the 50-CDs/rGO supercapacitor retained a high cycling stability after 1000 cycles in 1 M H_2_SO_4_. Overall, the specific capacitance of rGO was significantly improved by the addition of CDs, and the resulting electroactive material is a good candidate for supercapacitor applications.

## Supplementary Materials

The supplementary materials are available online at http://www.mdpi.com/2079-4991/6/11/212/s1.

## References

[1] Lu W., Qu L., Henry K., Dai L. (2009). High performance electrochemical capacitors from aligned carbon nanotube electrodes and ionic liquid electrolytes. J. Power Sources.

[2] Chen Z., Weng D., Wang X., Cheng Y., Wang G., Lu Y. (2012). Ready fabrication of thin-film electrodes from building nanocrystals for micro-supercapacitors. Chem. Commun..

[3] Sheng K., Sun Y., Li C., Yuan W., Shi G. (2012). Ultrahigh-rate supercapacitors based on eletrochemically reduced graphene oxide for ac line-filtering. Sci. Rep..

[4] Mao S., Lu G., Chen J. (2015). Three-dimensional graphene-based composites for energy applications. Nanoscale.

[5] Weitz R.T., Yacoby A. (2010). Nanomaterials: Graphene rests easy. Nat. Nanotechnol..

[6] Cerf A., Alava T., Barton R.A., Craighead H.G. (2011). Transfer-printing of single DNA molecule arrays on graphene for high-resolution electron imaging and analysis. Nano Lett..

[7] Ali A., Ali K., Kwon K.-R., Hyun M.Y. (2014). Electrohydrodynamic atomization approach to graphene/zinc oxide film fabrication for application in electronic devices. J. Mater. Sci. Mater. Electron..

[8] Chen K., Chen L., Chen Y., Bai H., Li L. (2012). Three-dimensional porous graphene-based composite materials: Electrochemical synthesis and application. J. Mater. Chem..

[9] Qian T., Yu C., Wu S., Shen J. (2013). A facilely prepared polypyrrole-reduced graphene oxide composite with a crumpled surface for high performance supercapacitor electrodes. J. Mater. Chem. A.

[10] Gao Z., Wang F., Chang J., Wu D., Wang X., Wang X., Xu F., Gao S., Jiang K. (2014). Chemically grafted graphene-polyaniline composite for application insupercapacitor. Electrochim. Acta.

[11] Jin L., Sun D., Zhang J.-R. (2012). Electropolymerization of the 3,4-ethylenedioxythiophene on the graphene nanosheet in ionic liquid for supercapacitors. Chin. J. Inorg. Chem..

[12] Patila U.M., Sohna J.S., Kulkarnia S.B., Parka H.G., Junga Y., Guravb K.V., Kimc J.H., Jun S.C. (2014). A facile synthesis of hierarchical α-MnO_2_ nanofibers on 3D-graphene foam for supercapacitor application. Mater. Lett..

[13] Liu H., Zhang J., Zhang B., Shi L., Tan S., Huang L. (2014). Nitrogen-doped reduced graphene oxide-Ni(OH)_2_-built 3D flower composite with easy hydrothermal process and excellent electrochemical performance. Electrochim. Acta.

[14] Zhu S., Song Y., Zhao X., Shao J., Zhang J., Yang B. (2015). The photoluminescence mechanism in carbon dots (graphene quantum dots, carbon nanodots, and polymer dots): Current state and future perspective. Nano Res..

[15] Li Y., Zhao Y., Cheng H., Hu Y., Shi G., Dai L., Qu L. (2012). Nitrogen-doped graphene quantum dots with oxygen-rich functional groups. J. Am. Chem. Soc..

[16] Wang Y., Hu A. (2014). Carbon quantum dots: Synthesis, properties and applications. J. Mater. Chem. C.

[17] Fernando K.A.S., Sahu S., Liu Y., Lewis W.K., Guliants E.A., Jafariyan A., Wang P., Bunker C.E., Sun Y.-P. (2015). Carbon quantum dots and applications in photocatalytic energy conversion. ACS Appl. Mater. Interfaces.

[18] Huang C., Hong Y., Yan X., Xiao L., Huang K., Gu W., Liu K., Shi W. (2016). Carbon quantum dots decorated hollow In_2_S_3_ microspheres with efficient visible-light-driven photocatalytic activities. RSC Adv..

[19] Yu J., Xu C., Tian Z., Lin Y., Shi Z. (2015). Facilely synthesized N-doped carbon quantum dots with high fluorescent yield for sensing Fe^3+^. New J. Chem..

[20] Yan R., Wu H., Zheng Q., Wang J., Huang J., Ding K., Guo Q., Wang J. (2014). Graphene quantum dots cut from graphene flakes: High electrocatalytic activity for oxygen reduction and low cytotoxicity. RSC Adv..

[21] Niu J., Gao H. (2014). Synthesis and drug detection performance of nitrogen-doped carbon dots. J. Lumin..

[22] Zhu Y., Ji X., Pan C., Sun Q., Song W., Fang L., Chen Q., Banks C.E. (2013). A carbon quantum dot decorated RuO_2_ network: Outstanding supercapacitances under ultrafast charge and discharge. Energy Environ. Sci..

[23] Liu W.-W., Feng Y.-Q., Yan X.-B., Chen J.-T., Xue Q.-J. (2013). Superior micro-supercapacitors based on graphene Quantum Dots. Adv. Funct. Mater..

[24] Shen B., Lang J., Guo R., Zhang X., Yan X. (2015). Engineering the electrochemical capacitive properties of micro-supercapacitors based on graphene quantum dots/MnO_2_ using ionic liquid gel electrolytes. ACS Appl. Mater. Interfaces.

[25] Mondal S., Rana U., Malik S. (2015). Graphene quantum dot-doped polyaniline nanofiber as high performance supercapacitor electrode materials. Chem. Commun..

[26] Liu W.-W., Yan X.-B., Chen J.-T., Xue Q.-J. (2013). Novel and high-performance asymmetric micro-supercapacitors based on graphene quantum dots and polyaniline nanofibers. Nanoscale.

[27] Zhu S., Meng Q., Wang L., Zhang J., Song Y., Jin H., Zhang K., Sun H., Wang H., Yang B. (2013). Highly photoluminescent carbon dots for multicolor patterning, sensors, and bioimaging. Angew. Chem. Int. Ed..

[28] Peng J., Gao W., Gupta B.K., Liu Z., Aburto R.R., Ge L., Song L., Alemany L.B., Zhan X., Gao G. (2012). Graphene quantum dots derived from carbon fibers. Nano Lett..

[29] Wan W., Li L., Zhao Z., Hu H., Hao X., Winkler D.A., Xi L., Hughes T.C., Qiu J. (2014). Graphene oxide: Ultrafast fabrication of covalently cross-linked multifunctional graphene oxide monoliths. Adv. Funct. Mater..

[30] Sbirrazzuoli N., Mititelu-Mija A., Vincent L., Alzina C. (2006). Isoconversional kinetic analysis of stoichiometric and off-stoichiometric epoxy-amine cures. Thermochim. Acta.

[31] Dai B., Fu L., Liao L., Liu N., Yan K., Chen Y., Liu Z. (2011). High-quality single-layer graphene via reparative reduction of graphene oxide. Nano Res..

[32] Todorova N., Giannakopoulou T., Boukos N., Vermisoglou E., Lekakou C., Trapalis C. (2016). Self-propagating solar light reduction of graphite oxide in water. Appl. Surf. Sci..

[33] Vermisoglou E.C., Giannakopoulou T., Romanos G., Giannouri M., Boukos N., Lei C., Lekakou C., Trapalis C. (2015). Effect of hydrothermal reaction time and alkaline conditions on the electrochemical properties of reduced graphene oxide. Appl. Surf. Sci..

[34] Beidaghi M., Wang C. (2012). Supercapacitors: Micro-supercapacitors based on interdigital electrodes of reduced graphene oxide and carbon nanotube composites with ultrahigh power handling performance. Adv. Funct. Mater..

[35] Wen X., Zhang D., Shi L., Yan T., Wang H., Zhang J. (2012). Three-dimensional hierarchical porous carbon with a bimodal pore arrangement for capacitive deionization. J. Mater. Chem..

[36] Fan Z., Yan J., Wei T., Zhi L. (2011). Asymmetric supercapacitors based on graphene/MnO_2_ and activated carbon nanofiber electrodes with high power and energy density. Adv. Funct. Mater..

[37] Li R., Ren X., Zhang F., Du C., Liu J. (2012). Synthesis of Fe_3_O_4_@SnO_2_ core-shell nanorod film and its application as a thin-film supercapacitor electrode. Chem. Commun..

[38] Stoller M.D., Park S., Zhu Y., An J., Ruoff R.S. (2008). Graphene-based ultracapacitors. Nano Lett..

[39] Wang Y., Shi Z., Huang Y., Ma Y., Wang C., Chen M., Chen Y. (2009). Supercapacitor devices based on graphene materials. J. Phys. Chem. C.

[40] Sun H., Cao L., Lu L. (2012). Bacteria promoted hierarchical carbon materials for high-performance supercapacitor. Energy Environ. Sci..

[41] Niu Z., Luan P., Shao Q., Dong H., Li J., Chen J., Zhao D., Cai L., Zhou W., Chen X. (2012). A “skeleton/skin” strategy for preparing ultrathin free-standing single-walled carbon nanotube/polyaniline films for high performance supercapacitor electrodes. Energy Environ. Sci..

[42] Ji H., Zhang L., Pettes M.T., Li H., Chen S., Shi L., Piner R., Ruoff R.S. (2012). Ultrathin graphite foam: A three-dimensional conductive network for battery electrodes. Nano Lett..

[43] Zhang F., Tang Y., Liu H., Ji H., Jiang C., Zhang J., Zhang X., Lee C.-S. (2016). Uniform incorporation of flocculent molybdenum disulfide nanostructure into three-dimensional porous graphene as an anode for high-performance lithium ion batteries and hybrid supercapacitors. ACS Appl. Mater. Interfaces.

[44] Zhou Q., Zhao Z., Zhang Y., Meng B., Zhou A., Qiu J. (2012). Graphene sheets from graphitized anthracite coal: Preparation, decoration, and application. Energy Fuels.

