# Supplementary material for: Electrochemical and Capacitive Properties of Carbon Dots/Reduced Graphene Oxide Supercapacitors
Source: Nanomaterials (Basel). 2016 Nov 14;6(11):212. doi: 10.3390/nano6110212 (PMC5245759; doi:10.3390/nano6110212)
Supplement: Supplementary file 1 [file nanomaterials-06-00212-s001.pdf]

Supplementary Materials: Electrochemical and Capacitive Properties of Carbon Dots/Reduced Graphene Oxide Supercapacitors

Yong-Qiang Dang, Shao-Zhao Ren, Guoyang Liu, Jiangtao Cai, Yating Zhang and Jieshan Qiu

Table S1. The specific capacitances of the rGO and various CDs/rGO electrodes at different current densities.

Number	Electrode Material	*C* (F/g)		
		i = 0.5 A/g	*i* = 1 A/g	*i* = 2 A/g
1	rGO	121.6	79.8	54.0
2	50-CDs/rGO (1:1)	137.8	125.8	118.0
3	50-CDs/rGO (2:1)	166.4	148.8	131.5
4	50-CDs/rGO (5:1)	211.9	186.5	155.3
5	50-CDs/rGO (8:1)	124.1	105.0	87.8
6	50-CDs/rGO (10:1)	105.8	98.0	87.3
7	20-CDs/rGO (5:1)	189.7	158.8	139.8
8	40-CDs/rGO (5:1)	192.7	167.6	154.5
9	60-CDs/rGO (5:1)	49.7	41.9	36.8
10	80-CDs/rGO (5:1)	18.2	15.9	14.8

© 2016 by the authors. Submitted for possible open access publication under the terms and conditions of the Creative Commons Attribution (CC-BY) license (http://creativecommons.org/licenses/by/4.0/).
